# Acute Presentation of Lumbar Spinal Schwannoma Due to Torsion: A Case Report

**DOI:** 10.7759/cureus.586

**Published:** 2016-04-25

**Authors:** Winward Choy, Ryan Khanna, Thomas C Ortmeier, Gino G Tapia-Zegarra, Timothy E Lindley, Zachary A Smith, Nader S Dahdaleh

**Affiliations:** 1 Neurosurgery, Feinberg School of Medicine Northwestern University; 2 Department of Neurosurgery, Feinberg School of Medicine Northwestern University; 3 Department of Pathology, Sanford Health; 4 Department of Internal Medicine, Sanford Health; 5 Department of Neurosurgery, Sanford Health

**Keywords:** spine, schwannoma, torsion

## Abstract

Although schwannomas are common spinal tumors with insidious presentations, acute neurological deterioration is an extremely rare manifestation that can occur in the setting of tumor torsion and infarction. The present case reports an unusual presentation of a spinal schwannoma that underwent torsion and infarction. A 65-year-old male presented initially with acute radicular pain progressing to cauda equina syndrome and confusion. MRI of the lumbar spine revealed an intradural extramedullary lesion at the level of L1/L2 measuring 1.1x0.9 cm. Intraoperatively, a reddish mass was seen caudally twisted around itself. Gross total resection was achieved with a final diagnosis of schwannoma with areas of infarction. At his six week follow up clinical visit, the patient was asymptomatic and his neurological exam was normal. The neurosurgeon should be aware of such atypical radiographic and clinical presentation amongst the spectrum of clinical manifestation of these nerve sheath tumors.

## Introduction

Spinal schwannomas are slow-growing tumors that account for one-fifth of all spinal tumors. Their presentation is usually insidious, and most patients complain of extremity pain [1]. An acute presentation of a spinal schwannoma from torsion is exceptionally rare, with only four other reports in the literature [2-5]. Here, we report an unusual manifestation of a spinal schwannoma that underwent torsion and infarction, resulting in acute radicular pain progressing to cauda equina syndrome and confusion. 

## Case presentation

A 65-year-old male presented to the emergency room with the acute onset of low back and right lower extremity pain, urinary retention, and confusion. Six days prior, the patient complained of low back pain with radiation to the right buttock, groin, and lower extremity. He was diagnosed as having sciatica at an outside emergency room and discharged with oral pain medications. At presentation, the pain was poorly controlled and complicated with progressive confusion and urinary retention. On exam, the patient was afebrile with normal vitals. He was awake but only oriented to person. Cranial nerves, deep tendon reflexes, and strength and sensation in the upper and lower extremities were all normal. Informed patient consent was obtained for treatment.

**Figure 1:**
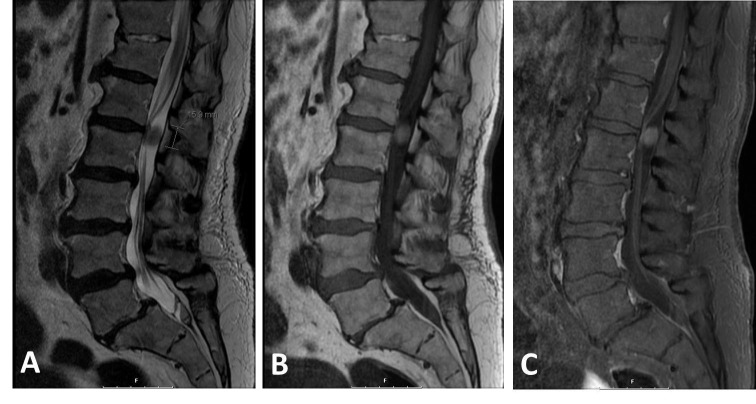
Magnetic resonance imaging (MRI) of spinal schwannoma A: Sagittal T2-weighted sequence showing an intradural extramedullary lesion that is well-circumscribed and has low signal intensity. B: The lesion has a high signal intensity on the T1-weighted sequence and (C) mildly enhances to contrast.

An enhanced magnetic resonance imaging of the brain was normal. Complete blood count revealed a white blood cell count of 7,800 /uL. The erythrocyte sedimentation rate was 22 mm/hr and C-reactive protein was < 0.5 mg/L. Cerebrospinal fluid (CSF) from a lumbar puncture revealed a glucose of 85 mg/dL, protein of 195 mg/dL, RBC count of 185 /uL, and WBC of 2,530 /uL. The cell differential was 2% lymphocytes and 90% neutrophils. Microscopic examination revealed no organisms. CSF was also sent for herpes simplex virus PCR and enterovirus PCR, and serum was sent for West Nile IgM, RPR, and HIV, all of which were negative. An enhanced MRI of the lumbar spine revealed an intradural extramedullary lesion at the level of L1/L2, measuring 1.1 x 0.9 cm. The T2-weighted sequence showed low signal attenuation and the T1-weighted sequence showed high signal attenuation with minimal contrast enhancement (Figure 1).

**Figure 2:**
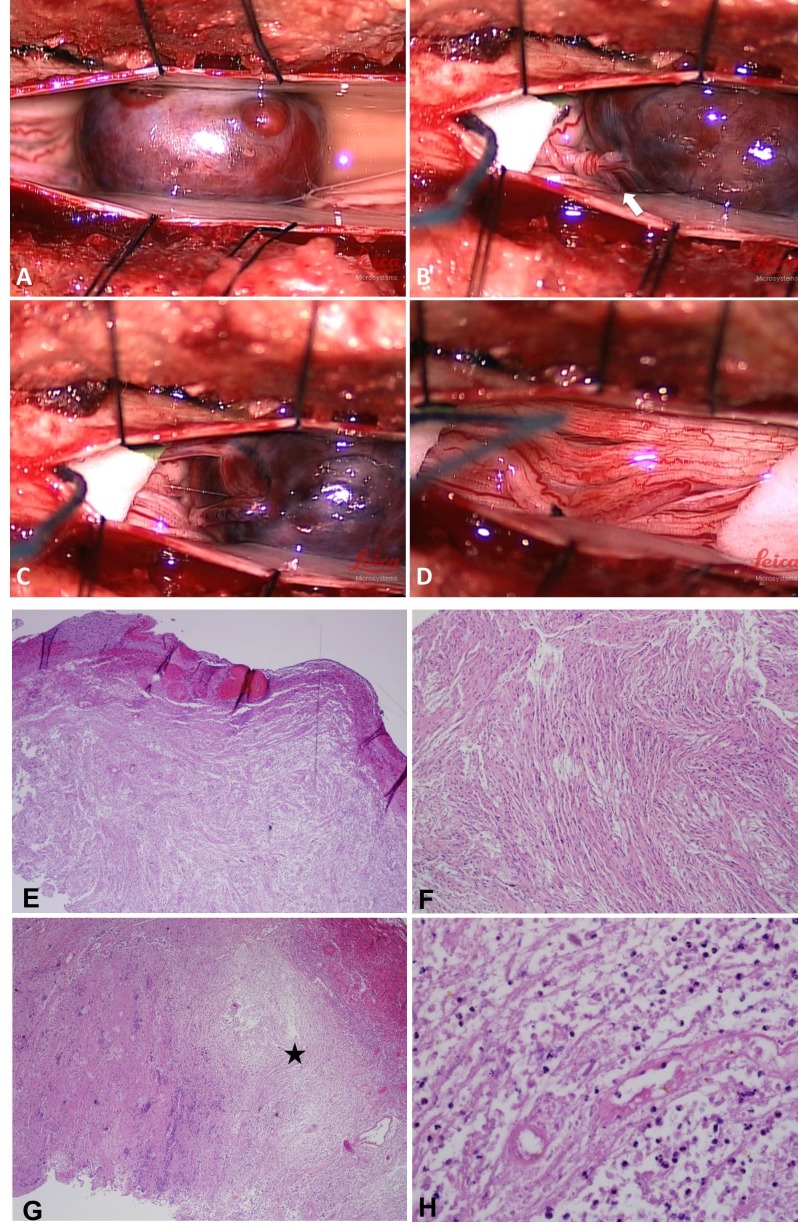
Intraoperative composite image and histopathology A: The infarcted schwannoma had a dark reddish color. B: The tumor had twisted around itself and wrapped nerve roots around its originating nerve root (Arrow). C: View after the tumor was “unrolled” to detangle nerve roots. D: The tumor was resected en bloc. E: Low power view of encapsulated schwannoma with vascular congestion, a viable portion of the tumor, 40x. F: Medium power view of the schwannoma with spindle cells, fasciculated to some extent, with degenerative change, 100x. G: Low power view of the necrotic area in schwannoma, 40x (asterisk). H: High power view of necrosis and acute inflammation in schwannoma.

Empiric antibiotics were started pending final cultures, as the results of the lumbar puncture were suggestive of an inflammatory process and possible meningitis. Despite antimicrobial therapy, the patient’s confusion, pain, and urinary retention persisted. Blood and CSF cultures obtained on admission did not demonstrate any bacterial growth. Due to his persistent symptoms and failure to reach a conclusive diagnosis, the decision was made to explore the lesion within the lumbar spinal canal. An L1-L2 laminoplasty was performed exposing the dura. The dura then was incised, revealing an intradural well-circumscribed reddish mass. The caudal area of the lesion appeared necrotic and dark purple in color. It was noticed that the mass was caudally twisted around itself and wrapped nerve roots around its original nerve root. The tumor was “unrolled” to detangle nerve roots. Intraoperative triggered electromyography was performed to test the nerve root entering the mass and no response was identified. The nerve root was coagulated with bipolar cautery above and below the mass and divided. The mass was removed *en bloc* (Figure 2). The dura then was approximated. The lamina was secured back into position with small titanium plates. The wound was closed in multiple layers. The pathology of the tumor was a schwannoma with areas of infarction (Figures 2E-2H). The patient was admitted to the regular inpatient unit. His pain improved immediately after surgery. By the next day, the patient’s mental status had normalized, and he was no longer suffering from urinary retention. He was discharged on postoperative day two. As gross total resection was achieved, no other adjuvant therapy was pursued. At his six-week follow-up clinical visit, the patient was asymptomatic and his neurological exam was normal.

## Discussion

Within the spine, schwannomas are most commonly located in the cauda equina region, followed by the thoracic spine. Their clinical presentation is typically insidious, at times lasting for months or even years. The presentation is variable, from no symptoms to pain, radiculopathy, motor deficits, and cauda equina syndrome. The present case highlights an unusual presentation of abrupt neurological deterioration and mental status changes, which are extremely rare manifestations of spinal schwannoma. The reported mental status changes in spinal tumors, in general, are attributed to hydrocephalus in upper cervical tumors obstructing cerebrospinal fluid (CSF) pathways. These tumors are most commonly intramedullary and are located in the upper cervical spine extending into the brainstem, and often times are associated with syringomyelia and syringobulbia [1].

Our case demonstrates an unusual clinical presentation of spinal schwannoma with acute radiculopathy. This rapidly progressed over days to severe mental status changes as well as cauda equina syndrome manifesting as urinary retention. A lumbar puncture was done on presentation to evaluate for meningitis as a possible cause of the acute mental status changes. The findings were congruent with aseptic meningitis with an elevated white blood cell count, along with normal glucose. Moreover, the persistent confusion was likely attributed to aseptic meningitis. MR imaging demonstrated a minimally enhancing tumor that was congruent with intraoperative and, later, histopathological findings of an infarcted schwannoma. A standard surgical approach of laminectomy, dural opening, and microsurgical resection of the tumor was performed. The nerve sheath tumor was twisting the associated nerve roots. The tumor was easily unwound in the opposite direction and then resected, sparing all functional nerve roots of the cauda equina. Aseptic meningitis has never been reported in the context of intraspinal schwannoma. In this case, it might explain the acute mental status changes observed in our patient.

A review of the literature found four other reports of spinal schwannoma undergoing torsion (Table 1) [2-5]. Inclusive of the present case, mean age at presentation for these five patients was 47.6 years (range: 16-65 years), and all cases were male. In four of the five cases, the patients presented with severe pain located in the back [3], rectal area [5], or lower extremities [4]. While the precise etiology is unclear, authors have attributed the pain to vascular irritation, or mechanical distortion and activation of pain fibers within the affected nerve following torsion [4]. Other symptoms include urinary retention [3], lower extremity numbness and weakness [2, 4], and acute symptoms of cord compression [2]. Lesions were identified mainly within the lumbar spine with four of the cases at L1-L3 [3-5], and one case at T3-T4 [2]. Given the abruptness of presentation in the setting of a mass lesion, the differential diagnoses include infection, abscess, or hemorrhage of the tumor [4].

**Table 1:** Summary of Reported Cases of Spinal Schwannoma Torsion Acronyms: M = male, Sx = symptoms, L = Left, R = Right, LE = lower extremity, EOR = extent of resection, GTR = gross total resection

Study	Age	Sex	Presenting Symptoms	Location	Size (cm)	EOR	Histopathology	Outcomes
Kornel, et al. [5] (1988)	58	M	Acute and severe rectal pain	L1-L2	-	GTR	Necrosis, hemorrhage, and inflammation	Resolution of preoperative Sx
Shrier, et al. [2] (1995)	37	M	Fever, chills, L pleuritic chest pain, numbness below T4, and 4/5 strength in LE	T3-T4	1.8 x 1.2	-	Acute infarction, edema, necrosis, and thrombosis	-
Khoshyomn, et al. [3] (2002)	16	M	acute low back pain, urinary retention	L3	-	GTR	Infarction	-
Jenkins, et al. [4] (2015)	62	M	Abrupt pain and numbness in L4 distribution. 4/5 weakness with R dorsiflexion	L2-L3	1.8 x 1.2x1	-	Myxoid subtype; hemorrhage	Resolution of preoperative Sx, 11 yr follow-up
Choy, et al. (2015)	65	M	Acute low back pain, RLE pain, urinary retention, and confusion	L1-L2	1.1 x .09	GTR	Infarction	Resolution of preoperative Sx, 6-week follow-up

Intraoperative and histopathological findings were similar to the present case. Reports often describe the identification of torsion of a well-circumscribed reddish brown lesion [4-5], with signs of infarction [3] or vascular supply compression [4]. Hemorrhage was reported in two cases, either with or without necrosis [5]. Other histopathological features included signs of arterial thrombosis, coagulation necrosis, edema, and inflammation [2, 4]. Gross total resection (GTR) was achieved in all cases reporting the extent of resection, as reported in the present case. Patients uniformly recover well postoperatively with the resolution of pain upon long-term follow-up [4-5].

While the exact mechanism remains unclear, we suggest that at some point in time the tumor twisted mechanically and obstructed the blood supply (Figure 2). This, in turn, caused tumor infarction and necrosis as well as aseptic meningitis. This clinically then manifested as acute radiculopathy and confusion. The swelling and edema inside the tumor might also explain the abrupt neurological deterioration due to mass effect.

## Conclusions

Schwannomas are common spinal tumors with insidious presentations. However, the present case highlights an atypical manifestation characterized by an acute neurological deterioration in the setting of tumor torsion and infarction. Despite the acute presentation, resolution of the pain and neurological symptoms followed resection of the torsed schwannoma. The neurosurgeon should be aware of such an atypical radiographic and acute clinical presentation amongst the spectrum of clinical manifestations of these nerve sheath tumors. 
